# Radiation Shielding of Fiber Reinforced Polymer Composites Incorporating Lead Nanoparticles—An Empirical Approach

**DOI:** 10.3390/polym13213699

**Published:** 2021-10-27

**Authors:** Rabie A. Abu Saleem, Nisrin Abdelal, Ahmad Alsabbagh, Maram Al-Jarrah, Fatima Al-Jawarneh

**Affiliations:** 1 Nuclear Engineering Department, Jordan University of Science and Technology, P.O. Box 3030, Irbid 22110, Jordan; Ahalsabbagh@just.edu.jo (A.A.); maramahmad072@gmail.com (M.A.-J.); Fatima.jawa97@gmail.com (F.A.-J.); 2 Mechanical Engineering Department, Jordan University of Science and Technology, P.O. Box 3030, Irbid 22110, Jordan; nrabdelal@just.edu.jo

**Keywords:** fiber reinforced polymer composites, lead nanoparticles, shielding, attenuation coefficient, empirical derivation

## Abstract

In the present work, an empirical approach based on a computational analysis is performed to study the shielding properties of epoxy/carbon fiber composites and epoxy/glass fiber composites incorporating lead nanoparticle (PbNPs) additives in the epoxy matrix. For this analysis, an MCNP5 model is developed for calculating the mass attenuation coefficients of the two fiber reinforced polymer (FRP) composites incorporating lead nanoparticles of different weight fractions. The model is verified and validated for different materials and different particle additives. Empirical correlations of the mass attenuation coefficient as a function of PbNPs weight fraction are developed and statistically analyzed. The results show that the mass attenuation coefficient increases as the weight fraction of lead nanoparticles increases up to a certain threshold (~15 wt%) beyond which the enhancement in the mass attenuation coefficient becomes negligible. Furthermore, statistical parameters of the developed correlations indicate that the correlations can accurately capture the behavior portrayed by the simulation data with acceptable root mean square error (RMSE) values.

## 1. Introduction

The utilization of radiation has been steadily growing over the last decades in a variety of fields including medical, industrial and agricultural fields. Despite the immense benefits of radiation, it has the potential to pose a significant safety hazard for human health and the environment. There are three fundamental concepts pursued for better protection against radiation; decreasing the exposure time as much as possible, increasing the distance between the radiation source and the entity of interest and using a shielding material to physically separate the entity of interest from radiation. These three concepts are implemented as much as practically possible to reduce the total absorbed dose due to radiation exposure. This is referred to as the ALARA (as low as reasonably achievable) principle [1].

The performance of a material as a radiation shield is usually assessed by its capacity to halt the penetration of the incident radiation through different interaction mechanisms. Gamma radiation, characterized by high penetration power, interacts with matter by three different processes, namely, photoelectric absorption, Compton scattering and pair production. The probability of each interaction to occur depends on the energy of the incident gamma radiation and the composition of the shielding material. Photoelectric absorption is the predominant process for gamma radiations with low energies interacting with materials of high atomic number. For gamma radiations with high energies, pair production becomes the predominant process. The bulk behavior of gamma interaction with the shield material is characterized by the linear attenuation coefficient (*µ*) which depends on both the energy of the incident radiation and the characteristics of the material. A desirable shielding material is capable of attenuating gamma radiation with minimal alteration to its mechanical, thermal and electrical properties as well as its chemical and physical stability. All in all, several factors should be taken into account when a radiation shield is designed, this includes the type of radiation and its energy level, radiation intensity, material cost and the diversity of material properties including weight, toxicity and environmental compatibility [1,2,3]. Concrete, lead and bismuth are among the commonly used material for shielding against gamma radiation.

Research studies on developed materials for radiation shielding applications have been boosted due to their imperative role in the advanced technologies that use ionizing radiation such as radiology, nuclear medicine, advanced material characterization and controlled modification of the properties of many materials. This diverse range of functions needs advanced materials to be used for manufacturing protective structures to protect humans and the environment from the harmful effects of ionizing radiation. Alshahrani et al. investigated the radiation shielding properties of newly developed high Fe content amorphous alloys. They reported the shielding capacity per unit thickness of the investigated alloys within the photon energy spectrum considered [4]. From a different perspective, Tishkevich et al. studied designing shielding materials to protect the critical elements and blocks of the electronic products and semi-conductor units that work in elevated radiation environments. They particularly studied the structure and attenuation coefficients of the WCu composite material when used in electron and proton radiation environments, and they showed that the use of WCu composite materials offers a very attractive alternative to lead (Pb), in terms of protection against ionizing radiation, as an environmentally friendly material and from the point of view of mass-dimensional parameters [5]. Kara et al. evaluated the shielding properties of fabricated dolomite doped glasses for gamma-rays. They showed that dolomite additive improves the gamma protecting capacity of lithium borate glasses. As a result, it was concluded that a glass sample with the highest dolomite additive content exhibits better efficiency in terms of radiation shielding [6]. Researchers have also investigated enhancing the shielding properties for materials with well-known superior shielding properties, such as concrete. Aygün et al. investigated new chromium ore based heavy concrete containing different types of minerals. They showed that concretes with additives and aggregates have better, gamma-ray and neutron, shielding features in comparison with standard concrete and some heavy types of concrete [7].

Composite materials are widely used in aircraft applications due to their competing superior properties in terms of weight, cost, dimensional stability, and dielectric strength [8,9,10,11]. For aircraft applications, the shielding properties of a material become a key factor due to the elevated level of cosmic radiation with increased altitude. For aviation applications, the cosmic radiation interacts with the earth’s atmosphere and produces secondary particles including protons, neutrons, electrons, positrons, and photons [12]. There have been several studies that focus on the effective dose received by the aircrews during their flights. One study was based on Monte Carlo simulations using FLUKA code performed to study the ability of the aircraft structure to shield against galactic cosmic rays [13].

Recent research studies explored a variety of composite materials as potential shielding materials, this includes epoxy/Pb_3_O_4_ composites [14], tungsten/epoxy composites [15], Gd_2_O_3_/epoxy composites [16], nano concrete composites [2,17], and composites of silicon resin with additives [3]. In general, there are two major phases constituting a composite material, a continuous phase characterized by low stiffness and a weak structure called the matrix phase and a stiffer and stronger phase called the reinforcement phase that can be continuous or discontinuous. Fiber reinforced polymer (FRP) composites constitute a family of materials that has been extensively studied for a variety of applications [18,19]. When these composites are used for radiation shielding, the alteration in their mechanical and structural properties becomes a key factor in determining their suitability. For FRP composites, the polymer component is more amenable to changes in its mechanical and structural properties [20,21]. Epoxy, characterized by good durability against gamma and neutron radiation compared to other polymers, has been widely studied as a matrix phase for FRP composites utilized in the field of radiation and nuclear applications [22,23]. Little research has been conducted to study the shielding properties of FRP composites with lead nanoparticle additives.

In a previous study, mass attenuation coefficients of silicon resin loaded with PbO, Bi_2_O_3_, and WO_3_ micro- and nanoparticles were calculated [3]. In that study, results for mass attenuation coefficients (*µ_m_*) from a Monto Carlo Simulation were validated against data from the National Institute of Standards and Technology (NIST). The results showed that mass attenuation coefficients for composites with nanoparticles filler were better than that of composites with microparticles. This was attributed to the fact that smaller particle size leads to more uniform distribution in the matrix and an increased surface to mass ratio. Furthermore, the results showed that the attenuation power of the composite increases as the weight percentage of the filler increases. Tekin et al. studied the influence of micro- and nanoparticle size for WO_3_ and Bi_2_O_3_ particle types on shielding properties of hematite-serpentine concrete (HSC) using MCNPX code [24]. The model was validated by comparing results for mass attenuation coefficients of HSC from the MCNPX model with those obtained from XCOM at different energies, and a good agreement between the two sets of results was observed. The result showed that mass attenuation coefficients of nanoparticles/HSC composites were better than those of microparticles/HSC composites. Moreover, mass attenuation results for Bi_2_O_3_/HSC composites were better than those of WO_3_/HSC composites. This is due to the fact that the density and the atomic number for bismuth (Bi) are higher than those for tungsten (W). Tekin et al. studied the effect of nano/micro-sized WO3 particles on mass attenuation coefficient for concrete using MCNPX code, the model was validated by calculating the mass attenuation coefficient for concrete using MCNPX model and comparing the result with the one from XCOM at different energies and he found a good agreement between the results. The results showed that the mass attenuation coefficient for nanoparticles was better than that for microparticles and the mass attenuation coefficient decreased as the energy of the radiation source increased [25]. In a separate study, Tekin et al. developed an MCNPX model to study the mass attenuation coefficient of lead doped with nano-sized barite (BaSO_4_) [26]. Results from the MCNPX model were benchmarked against standard XCOM data at different radiation energies. The results of MCNPX simulations showed that the mass attenuation coefficient of lead was improved upon the addition of nano-sized barite, furthermore, the mass attenuation coefficient decreased as the energy of the incident gamma radiation was increased.

In another study, Kazemi et al. developed an MCNPX model to study the shielding properties for novel polyvinyl alcohol (PVA)/WO_3_ composite using micro- and nanosized WO_3_ particles [27]. The model was validated by comparing mass attenuation coefficients of aluminum from the National Institute of Standards and Technology (NIST) tables to those calculated by the MCNPX model at 0.662 MeV incident radiation energy. It was found that composites with WO_3_ nanoparticles exhibit mass attenuation coefficients higher than those of composites with WO_3_ microparticles. Finally, a summary of research results related to the shielding properties of composite materials is presented in Table 1.

In this study, an empirical approach is followed to derive mathematical correlations for the shielding properties of composite materials based on computational analysis. The analysis is performed to shed the light on the shielding properties of epoxy/carbon-fiber composites and epoxy/glass-fiber composites incorporating lead nanoparticles (PbNPs) taking into account the effect of varying the content of lead nanoparticles on the investigated properties. Fiber reinforced polymer (FRP) composites are chosen for this study because they are considered a promising candidate for structural applications due to their superior relevant properties such as lightweight, high specific strength, sound insulation, durability and corrosion resistance [8,9]. Furthermore, epoxy is considered as the matrix phase for this study because its excellent mechanical and chemical properties, good adhesive strength and dimensional stability make it a promising candidate for applications featuring severe radiation environments [15]. Moreover, superior properties of both carbon fiber and glass fiber, considered in this study, led to their wide utilization as reinforcement phases in composite materials. Such properties include high tensile strength, high modulus, high chemical resistance, and temperature resistance [8,28,29]. Finally, the high surface to volume ratio of nanoparticles leads to improving their mechanical and shielding properties, justifying the consideration of lead nanoparticles, a well-known gamma shielding material, for this research study [19].

### Monte Carlo N-Particle (MCNP5) Code

Monto Carlo simulations using the Monto Carlo N-Particle (MCNP5) code were considered as a source of data to derive mathematical correlations describing the shielding properties of composite materials with different fiber content and different weight fractions of lead nanoparticles.

Monte Carlo codes have been used extensively in studying the shielding properties of different composites incorporating nano-sized materials [27,35,36,37,38,39]. MCNP5 has been extensively used in the field of nuclear applications and radiology, more specifically radiation shielding and detection [1,40]. The code is generally used to solve the transport equations of photons, neutrons, and electrons, based on Monte Carlo methods where a particle is tracked until it is either absorbed or escaped the physical domain of interest. Every possible interaction of the particle is accounted for by assigning probability values (interaction cross-sections) and the overall behavior of the particles is recorded in an average sense. In other words, the expectation (mean) of the probability distribution function describing the behavior is calculated. Simulations with MCNP5 require preparation of an input file that contains a description of the problem including material compositions, geometry specifications, location and characteristics of the particles. Moreover, the type of output data required from such simulations is also specified in the input file (called tallies) and it is delivered to the user in a text output file. The input file of an MCNP5 simulation contains three main sections, namely, the cell cards section, where the shape and material content of the physical space of interest is defined, the surface cards section, where the surfaces used for the geometry definition of all cells are specified and the data card section, where all other aspects of the simulation are specified including the simulation mode, material isotopic content and type of output data required from the simulation (tallies). Each section is a collective of several text lines called cards. For more details on calculations theory and input file specifications the reader is advised to review MCNP5 manuals [40,41].

## 2. Methodology

In the present study, MCNP5 (version 5), is used in the photon transport mode (P-mode) to track the photon population of gamma radiation, in the form of collimated monoenergetic beams, inside the shielding material. Results from these simulations are used to determine linear attenuation coefficients of composite samples of different fiber contents and different weight fractions of lead nanoparticles.

**Figure 1:**
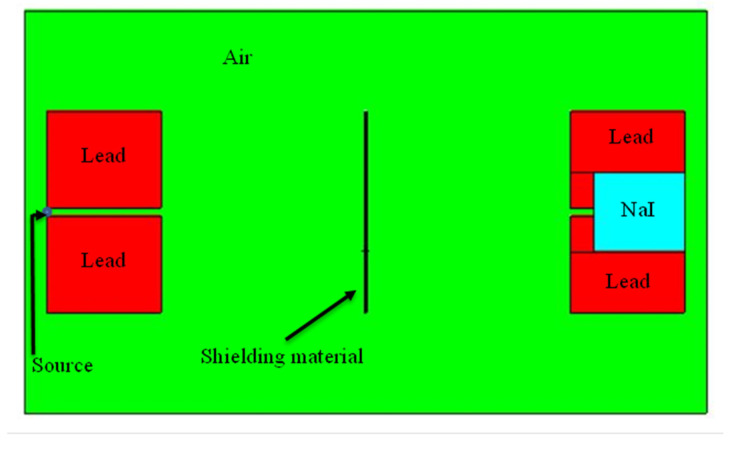
Schematic of the shielding test setup in the MCNP5 model.

A point isotropic source was defined using the source definition card (SDEF) with a source energy of 0.662 MeV corresponding to ^137^Cs source. The source is located at the center of the detection area emitting photons in a direction perpendicular to the composite sheet. Two sets of lead collimators were used, a source collimator consisting of two cuboids at the upper and lower sides of the source and a detector collimator located at the detector opposite to the source. Between the two sets of collimators, a shielding sheet of composite material is located with accurately specified dimensions. A cell flux tally (F4) is defined in the data cards section of the MCNP5 input file to estimate the total number of photons per unit area entering the cell that represents the NaI detector. Figure 1 shows a schematic of the physical domain defined for the MCNP5 simulation.

Cross-sectional data used in the simulation were obtained from the Evaluated Nuclear Data Files (ENDF/B-VI) library. For all simulations, the history cutoff card (NPS) was defined with a total of 10^6^ histories to be run in the problem and a relative statistical error set to less than 0.1%. Time, energy and particle weight cutoffs were all set to default values. Simulation real run time ranged between 1–3 h depending on the weight fraction of lead nanoparticles used. Simulations were performed using a machine with core i5-8250U CPU and 1.8 GHz speed. Upon completion of all simulations, a MATLAB script was developed to extract the desired data from the output files, process it and perform calculations for the determination of the shielding properties of the simulated material. Furthermore, statistical information was obtained from the output files to assess the precision of the results. All statistical parameters were satisfactory with relative error values less than 0.00035 and variance of the variance values less than 6.5 × 10^−6^, for all simulations.

The first step of this computational analysis was to validate the MCNP5 model for shielding materials of known properties. Mass attenuation coefficients of lead and aluminum with different radiation energies were considered. Results of the mass attenuation coefficient (*µ_m_*) from the MCNP5 model were compared to those obtained from the photon cross-sections database (XCOM) provided by the National Institute of Standards and Technology (NIST). MCNP5 results were also compared to calculation results based on the theoretical formulations presented in reference [42]. The formulations presented in this reference were in accordance with data obtained from the National Nuclear Data Center in Brookhaven National Laboratory. Moreover, the MCNP5 model was verified by comparing results of mass attenuation coefficients for silicon-resin/37.5 wt%WO_3_, silicon-resin/37.5 wt%PbO, and silicon-resin/37.5 wt%Bi_2_O_3_ composites to those reported by literature [3].

Finally, an MCNP5 model was developed to calculate linear attenuation coefficients of epoxy/fiber composites with different weight fractions of lead nanoparticles. Simulations were performed for a point isotropic source with a collimated and monoenergetic beam of 0.662 MeV energy. The composite material was modeled by a sheet of three alternating layers, two layers of epoxy-PbNPs mixture and one layer of fiber (carbon fiber or glass fiber). The simulated sheets mimic composite samples that are prepared by the well-known vacuum bagging process for fabricating fiber reinforced polymer (FRP) composites [43]. To achieve a 50:50 weight balance between fiber and epoxy, dimensions and compositions of the alternating layers were chosen such that the total mass of the two epoxy-PbNPs layers is similar to that of the fiber layer. Lead nanoparticles were uniformly distributed within the epoxy matrix using LATTICE and UNIVERSE features provided by MCNP5. The mixture was modeled by a lattice of epoxy cuboids each with a lead nanoparticle sphere of 80 nm diameter located at the center. Cuboid dimensions were changed for each weight fraction of lead nanoparticles to satisfy the aforementioned mass balance condition. Dimensions of the lattice cell for different weight fractions of lead nanoparticles along with corresponding material densities are reported in Table 2.

**Figure 2:**
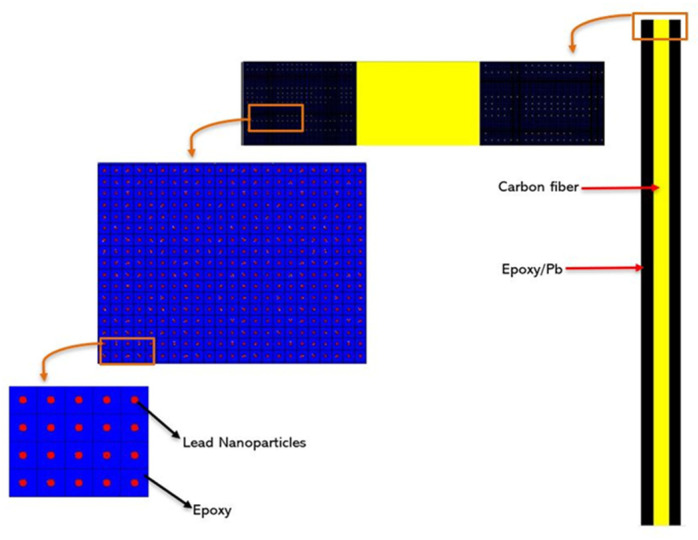
MCNP5 Cross-sectional screenshot for PbNPs blended into epoxy/Fiber composite.

A cross-sectional view of the composite sheet as modeled in MCNP5 is shown in Figure 2. The dimensions of the fiber sheet are set to 0.045 cm × 5 cm × 5 cm. For epoxy/PbNps sheets, the width and the height are set to 5 cm × 5 cm and the thickness was varied based on the weight fraction of lead nanoparticles. As for material densities, values of 1.7 g/cm^3^, 2.565 g/cm^3^, 1.1 g/cm^3^ and 11.35 g/cm^3^ were assigned for carbon fiber, glass fiber type E epoxy and lead nanoparticles, respectively. Detailed elemental composition of both, epoxy and glass fiber as defined in the MCNP5 model are provided in Table 3 and Table 4, respectively.

## 3. Results and Discussion

In this section, two sets of results are presented. Validation and verification results are presented in Section 3.1 and results on the effect of varying the weight fraction of PbNPs on the mass attenuation coefficient are presented in Section 3.2 in the form of empirically derived correlations for attenuation coefficients and mass attenuation coefficients.

### 3.1. Validation and Verification Results

**Figure 3:**
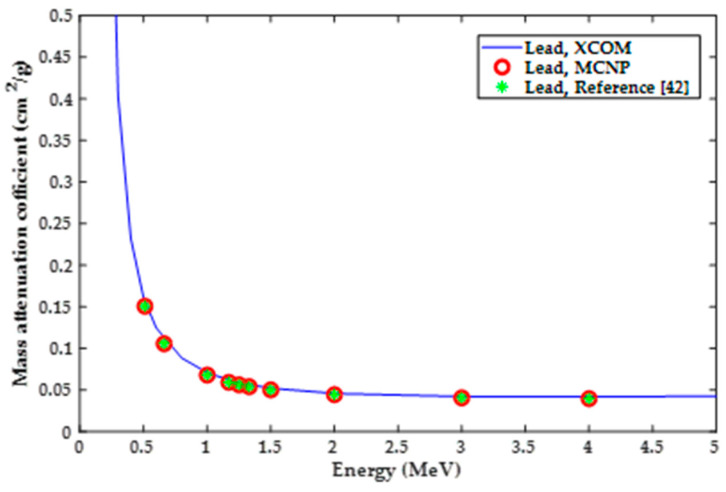
Validation results based on of lead mass attenuation coefficient.

**Figure 4:**
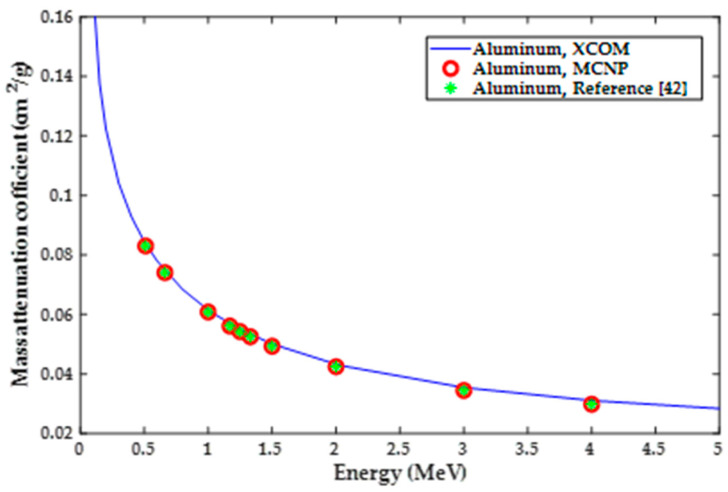
Validation results based on aluminum mass attenuation coefficient.

Mass attenuation coefficients for lead and aluminum for photon energies of 0.511, 0.662, 1, 1.17, 1.25, 1.33, 1.5, 2, 3, and 4 MeV were calculated using MCNP5 and compared to XCOM results provided by NIST and theoretical values based on the radiation shielding textbook [42]. Results of this validation for lead and aluminum are shown in Figure 3 and Figure 4, respectively. It can be seen that there is a good agreement between the three sets of results, XCOM, MCNP5 and theoretical results. This close agreement between the different sets of results was considered as a validation for the MCNP5 model for further simulation. Moreover, it can be seen from the figures that the mass attenuation coefficient tends to decrease with increasing the radiation energy, the decrease seems to be steeper for lead.

**Figure 5:**
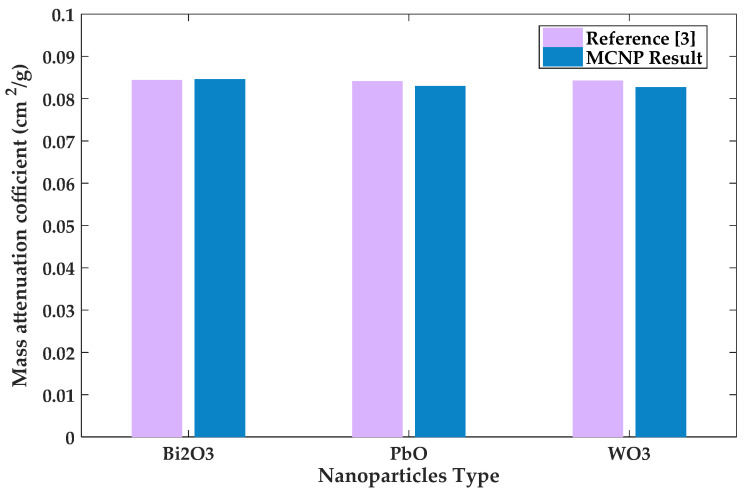
Verification results based on mass attenuation coefficients of silicon resin composites.

Additionally, verification of the MCNP5 model was done by comparing results from the model to those obtained in literature for composites with three different additives. By developing a new MCNP5 model containing three composite materials (silicon-resin/WO_3_, silicon-resin/PbO and silicon-resin/Bi_2_O_3_), the mass attenuation coefficient for each composite material was calculated for a photon energy of 0.6638 MeV and the concentration of nanoparticles equal to 37.5 wt%. Comparison between results of this analysis and Verdipoor’s results [3] are shown in Figure 5. The results from Verdipoor’s study entailed mass attenuation coefficients of 0.0843, 0.0841, and 0.0844 cm^2^/g, for silicon-resin/WO_3_, silicon-resin/PbO and silicon-resin/Bi_2_O_3_ composites, respectively. It can be seen that there is a good agreement between both sets of results with a maximum deviation of 1.9%.

### 3.2. Empirically Derived Correlations for the Shielding Properties of Composite Materials

MCNP5 simulations were performed to study the effect of the weight fraction of lead nanoparticles on the mass attenuation coefficient of composite materials. Results for epoxy/carbon fiber composites and epoxy/glass fiber composites are reported in Table 5. The calculated values of linear and mass attenuation coefficients for composites with weight fractions ranging from 0 wt% to 50 wt% at 0.662 MeV source energy are tabulated for increments of 2.5 wt%. Furthermore, the Mean Free Path (*MFP*), defined as the average distance between two successive photon interactions, was calculated using the following equation:

(1) $$MFP=\frac{1}{\mu }$$

Additionally, material thickness for which the intensity of the incident radiation is decreased by half, called the half-value layer (*HVL*), was calculated using the following equation:

(2) $$HVL=\frac{ln\left(2\right)}{\mu }$$

In the two equations above, *µ* is the linear attenuation coefficient of the material. For the two shielding properties, *MFP* and *HVL*, smaller values indicate higher rates of interaction, consequently, better shieling capabilities of the material.

Linear attenuation coefficients of glass fiber composites were found to be greater than those of carbon fiber composites, consequently, glass fiber composites exhibit smaller values for both *MFP* and *HVL*. This result is expected because the density of glass fiber is greater than the density of carbon fiber. This advantage in terms of density leads to an increased rate of interaction inside the material matrix, consequently, larger values of mass attenuation coefficients.

**Figure 6:**
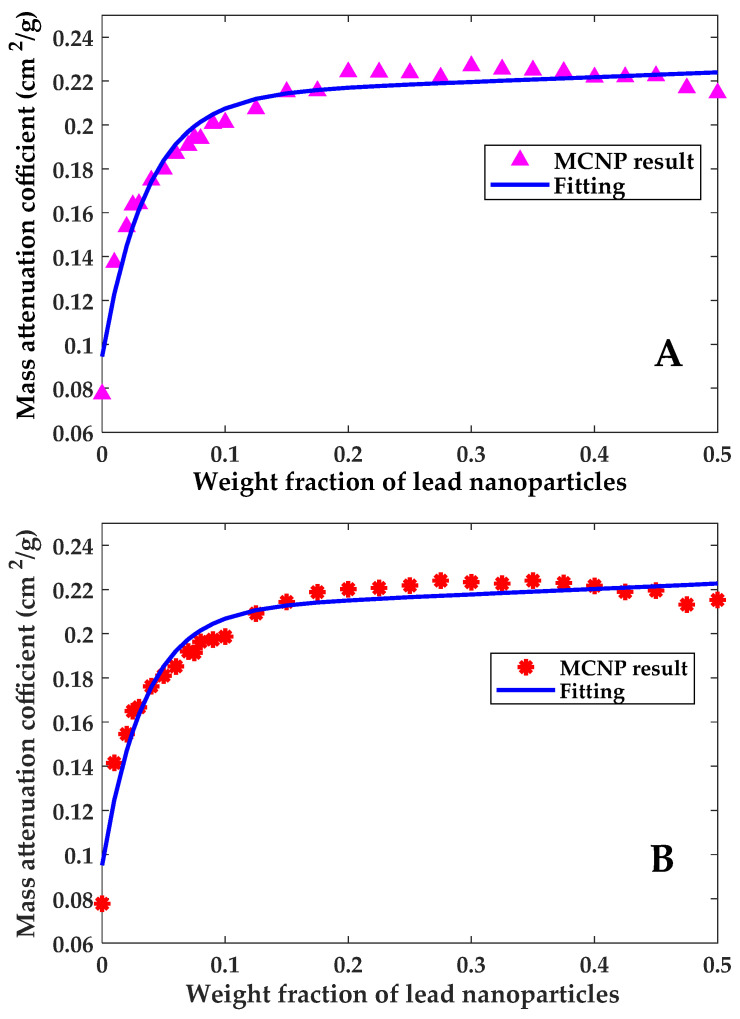
Computational results of *µ_m_* for (**A**) epoxy/CF-PbNPs (**B**) epoxy/GF-PbNPs.

As shown in Figure 6, results show that mass attenuation coefficients for both, carbon fiber composites and glass fiber composites increased as the lead weight fraction was increased up to a certain limit. Beyond that point (~15 wt%), the increment in mass attenuation coefficient becomes small even when the weight fraction of lead nanoparticles continued to increase (see Figure 6). Due to the negligible change in the mass attenuation coefficients of the two composites beyond this threshold value, a weight fraction of 15 wt% PbNPs is considered the optimal value for improved shielding properties of the two composites. By comparing the shielding properties of the two composites without PbNPs and those with 15 wt% of PbNPs, it can be concluded that the addition of lead nanoparticles leads to a reduction of ~64% in the mass required to shield against gamma radiation.

Furthermore, a comparison between the shielding properties of the two composites (with 15 wt% of PbNPs) to those of pure lead is presented in Table 6. It can be seen from Table 6 that the linear attenuation coefficient corresponding to the two composites is ~30–35% of that achieved by pure lead. Nevertheless, both composites show better behavior than pure lead in terms of the mass attenuation coefficient. Based on the data presented in Table 6, it can be concluded that the mass of either composite (with 15 wt% of PbNPs) required to shield against a given level of radiation is ~43% of the lead mass required to shield against the same level of radiation. The reduction in the mass required for shielding against radiation opens doors for a variety of applications where light weights and high strength are desired whereas high levels of radiation are encountered, examples of such applications include aviation and medical applications.

Curve fitting, based on a two-term exponential function, was performed in an attempt to find a mathematical correlation that correlates the mass attenuation coefficient to the weight fraction of lead nanoparticles. Fitting correlations for carbon fiber composites and glass fiber composites are expressed by Equation (3) and Equation (4), respectively.

(3) $$\mu_{m\left(CF\right)}\left(x\right)=0.2132e^{0.09857x}-0.1187e^{-27.22x}$$

(4) $$\mu_{m\left(GF\right)}\left(x\right)=0.2106e^{0.1114x}-0.1154e^{-29.31x}$$

In these equations, *x* is a number between 0 and 1 expressing the weight fraction of lead nanoparticles, *µ_m(CF)_* is the mass attenuation coefficient for epoxy/carbon fiber composite cm^2^/g and *µ_m(GF)_* is the mass attenuation coefficient for epoxy/glass fiber composite cm^2^/g.

The value of lead weight fraction beyond which the change in mass attenuation coefficient becomes negligible is around 15 wt%. At this weight fraction, the mass attenuation coefficient is calculated at 0.2144 cm^2^/g and 0.2127 cm^2^/g for carbon fiber composites and glass fiber composites, respectively.

**Figure 7:**
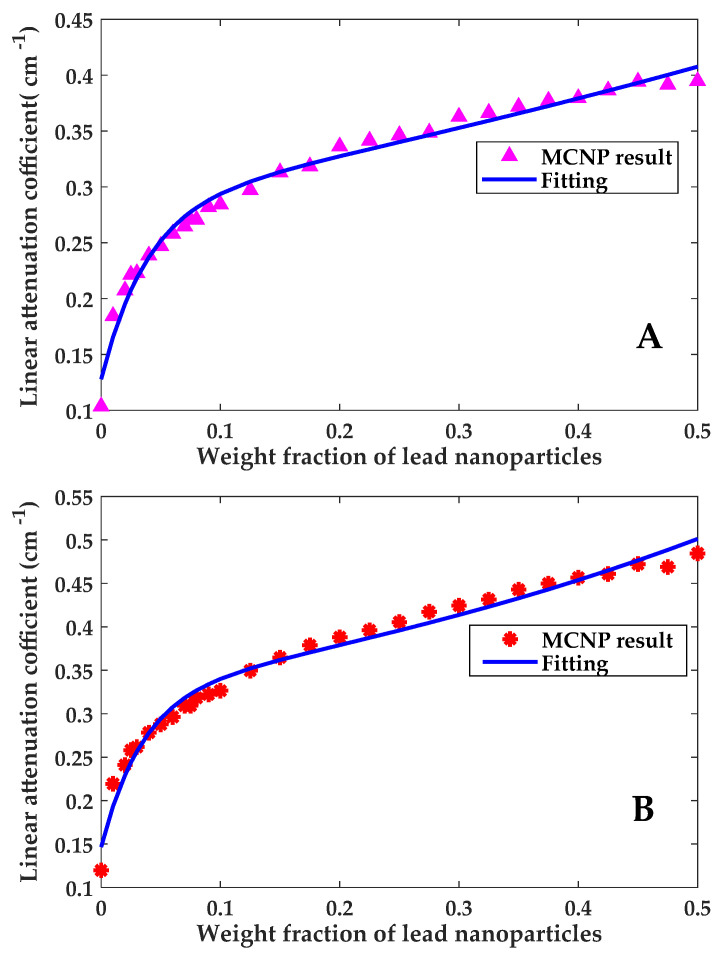
Computational results of *µ* for (**A**) epoxy/CF-PbNPs, (**B**) epoxy/GF-PbNPs.

Moreover, curve fitting was performed for the linear attenuation coefficient data presented in Table 5. Both the simulation data and the fitting curve are shown in Figure 7. Fitting correlations for carbon fiber composites and glass fiber composites are expressed by Equation (5) and Equation (6), respectively.

(5) $$\mu_{CF}\left(x\right)=0.2841e^{0.7217x}-0.1563e^{-25.89x}$$

(6) $$\mu_{GF}\left(x\right)=0.3218e^{0.8572x}-0.1737e^{-28.22x}$$

In these equations, *x* is a number between 0 and 1 expressing the weight fraction of lead nanoparticles, *µ_CF_* is the linear attenuation coefficient for epoxy/carbon fiber composite (cm^−1^) and *µ_GF_* is the linear attenuation coefficient for epoxy/glass fiber composite (cm^−1^).

To get an indication of the appropriateness of both correlations to capture the simulation data, statistical parameters for curve fitting were calculated and reported in Table 7. Statistical parameters in Table 7 give an indication that the correlations from curve fitting can accurately capture the behavior portrayed by the simulation data with acceptable root mean square error (RMSE) values.

## 4. Conclusions

A computational model based on Monte Carlo simulations was developed using MCNP5 to derive empirical correlations for the shielding properties of epoxy/fiber composites with different weight fractions of lead nanoparticles. After verifying and validating the model, it was implemented for fiber reinforced polymer composites incorporating lead nanoparticles and using two types of fiber, namely, carbon fiber and glass fiber. The results show that increasing the weight fraction of lead nanoparticles leads to increased values of mass attenuation coefficient. Nevertheless, there was a threshold value for PbNPs weight fraction beyond which the improvement in the mass attenuation coefficient becomes negligible. The threshold value of PbNP weight fraction was close to 15 wt%, for this weight fraction, the corresponding values for the mass attenuation coefficient were calculated at 0.2144 cm^2^/g for carbon fiber composites and 0.2127 cm^2^/g or glass fiber composites. Furthermore, the addition of lead nanoparticles led to a reduction in the *HVL* and *MFP* values, leading to decreased values for the material mass required to shield against gamma radiation. It was found that the addition of 15 wt% of PbNPs leads to a mass reduction of ~64% for the same level of shielding. Furthermore, the simulated composite samples with 15 wt% of lead nanoparticles showed better values for the mass attenuation coefficient compared to pure lead.

It can be concluded that the addition of lead nanoparticles to fiber reinforced composite materials is recommended for several applications, such as aviation applications, where high levels of radiation are expected, and light weights are required. Extensions to the work presented herein include, but are not limited to, experimental studies of the mechanical and shielding properties of composites incorporating different content of lead nanoparticles and comparing the results to the computational results of this study. Moreover, further simulation studies may be carried out for composites with different fiber content and different photon energies, shielding against other types of radiation, such as neutron and electron beam radiations, can also be investigated. Finally, other additives, such as tungsten and bismuth, can also be studied for their potential improvement on the shielding properties of fiber reinforced composite materials.

## Figures and Tables

**Table 1:** Shielding properties for different composite materials.

Shielding Material	Density (g/cm^3^)	Linear Attenuation Coefficient (cm^−1^)	Mass Attenuation Coefficient (cm^2^/g)	References
Lead	11.34	1.133	0.0999	[29]
Ordinary concrete	2.203	0.144	0.0654	[8]
Steel	8.020	0.433	0.0540	[8]
Epoxy/20 wt% PbO	-	0.091	-	
HD-PE/10 wt% PbO	1.051	0.105	0.0999	[30]
HD-PE/50 wt% PbO	1.652	0.189	0.1144	
Polyester/5 wt% PbO	1.2325	0.0997	0.0809	[31]
Polyester/10 wt% PbO	1.2891	0.114	0.0842	
Polyester/20 wt% PbO	1.4285	0.1264	0.0884	
Polyester/30 wt% PbO	1.6042	0.1422	0.0887	
Polyester/40 wt% PbO	1.855	0.1735	0.0935	
Polyester/50 wt% PbO	2.1721	0.206	0.0948	
Epoxy/50 wt% PbO	2.0034	0.17796	0.0888	[32]
Epoxy/70 wt% PbO	2.987	0.2723	0.0912	
Rubber/5 wt% Pb	-	0.00165	-	[33]
Rubber/20 wt% Pb	-	0.00221	-	
Rubber/50 wt% Pb	-	0.00298	-	
Rubber/75 wt% Pb	-	0.00478	-	
Epoxy/10 wt% PbO	1.26	0.1097	0.0871	[34]
Epoxy/30 wt% PbO	1.53	0.1414	0.0924	
Epoxy/50 wt% PbO	2.05	0.2005	0.0978	
Epoxy/70 wt% PbO	2.93	0.3091	0.1055	

**Table 2:** Lattice dimensions and composite densities used in MCNP5 model.

Lead Nanoparticles Weight Fraction %	Length of Lattice Cube Side ×10^−5^ cm	Density of the Composite Material g/cm^3^	
		With Carbon Fiber	With Glass Fiber
0	-	1.336	1.540
1	6.496	1.343	1.549
2	5.1401	1.351	1.559
2.5	4.7644	1.354	1.564
3	4.4765	1.358	1.569
4	4.0546	1.366	1.580
5	3.7521	1.373	1.590
6	3.5197	1.381	1.600
7	3.3328	1.389	1.611
7.5	3.2518	1.393	1.616
8	3.1774	1.397	1.622
9	3.0451	1.405	1.633
10	2.93	1.413	1.644
12.5	2.6976	1.434	1.672
15	2.5168	1.455	1.701
17.5	2.3698	1.477	1.731
20	2.2462	1.500	1.762
22.5	2.1397	1.524	1.795
25	2.0461	1.548	1.829
27.5	1.9627	1.573	1.864
30	1.8873	1.599	1.900
32.5	1.81814	1.625	1.938
35	1.755	1.653	1.977
37.5	1.6960	1.681	2.018
40	1.6408	1.711	2.061
42.5	1.5888	1.742	2.105
45	1.5395	1.773	2.152
47.5	1.4926	1.806	2.200
50	1.4477	1.840	2.251

**Table 3:** Elemental composition for epoxy (density is 1.1 g/cm^3^).

Element	Weight Percentage
Carbon	0.6421
Hydrogen	0.0669
Oxygen	0.2309
Chloride	0.0601

**Table 4:** Elemental composition for E-glass fiber (density is 2.565 g/cm^3^).

Element	Weight Percentage
Boron	0.022803
Oxygen	0.471950
Fluorine	0.004895
Sodium	0.007262
Magnesium	0.014759
Aluminum	0.072536
Silicon	0.247102
Potassium	0.008127
Calcium	0.143428
Titanium	0.004400
Iron	0.002739

**Table 5:** Computational results of linear and mass attenuation coefficients for composite materials.

Percentage of PbNPs wt%	Carbon Fiber				Glass Fiber			
	*µ_m_* (cm^2^/g)	*µ* (cm^−1^)	*HVL* (cm)	*MFP* (cm)	*µ_m_* (cm^2^/g)	*µ* (cm^−1^)	*HVL* (cm)	*MFP* (cm)
0	0.0775	0.1035	6.697	9.662	0.0778	0.1198	5.786	8.347
1	0.1373	0.1844	3.759	5.423	0.1415	0.2193	3.161	4.56
2	0.1536	0.2075	3.34	4.819	0.1545	0.2410	2.876	4.149
2.5	0.1635	0.2214	3.131	4.517	0.1650	0.2581	2.686	3.874
3	0.1640	0.2228	3.111	4.488	0.1667	0.2616	2.65	3.823
4	0.1747	0.2386	2.905	4.191	0.1761	0.2782	2.492	3.595
5	0.1799	0.2470	2.806	4.049	0.1810	0.2878	2.408	3.475
6	0.1869	0.2582	2.685	3.873	0.1851	0.2963	2.339	3.375
7	0.1906	0.2648	2.618	3.776	0.1920	0.3092	2.242	3.234
7.5	0.1942	0.2705	2.562	3.697	0.1913	0.3093	2.241	3.233
8	0.1937	0.2707	2.561	3.694	0.1963	0.3183	2.178	3.142
9	0.2007	0.2819	2.459	3.547	0.1973	0.3221	2.152	3.105
10	0.2011	0.2842	2.439	3.519	0.1986	0.3265	2.123	3.063
12.5	0.2073	0.2972	2.332	3.365	0.2091	0.3496	1.983	2.86
15	0.2151	0.3131	2.214	3.194	0.2144	0.3646	1.901	2.743
17.5	0.2155	0.3184	2.177	3.141	0.2188	0.3787	1.83	2.641
20	0.2241	0.3362	2.062	2.974	0.2201	0.3879	1.787	2.578
22.5	0.2240	0.3414	2.03	2.929	0.2206	0.3960	1.75	2.525
25	0.2237	0.3463	2.002	2.888	0.2218	0.4055	1.709	2.466
27.5	0.2217	0.3486	1.988	2.869	0.2240	0.4174	1.661	2.396
30	0.2269	0.3627	1.911	2.757	0.2233	0.4243	1.634	2.357
32.5	0.2254	0.3663	1.892	2.73	0.2226	0.4313	1.607	2.319
35	0.2249	0.3717	1.865	2.69	0.2239	0.4428	1.565	2.258
37.5	0.2242	0.3770	1.839	2.653	0.2229	0.4498	1.541	2.223
40	0.2218	0.3794	1.827	2.636	0.2216	0.4567	1.518	2.19
42.5	0.2219	0.3864	1.794	2.588	0.2189	0.4608	1.504	2.17
45	0.2223	0.3942	1.758	2.537	0.2195	0.4722	1.468	2.118
47.5	0.2168	0.3915	1.77	2.554	0.2131	0.4689	1.478	2.133
50	0.2145	0.3947	1.756	2.534	0.2152	0.4845	1.431	2.064

**Table 6:** Comparison of composites with 15 wt% of PbNPs to pure lead.

Material	Density (g/cm^3^)	*µ_m_* (cm^2^/g)	*µ* (cm^−1^)	*HVL* (cm)	*MFP* (cm)
Pure lead	11.29	0.0917	1.0404	0.666	0.961
CF-composite	1.455	0.2151	0.3131	2.214	3.194
GF-composite	1.701	0.2144	0.3646	1.901	2.743

**Table 7:** Statistical parameters for curve fitting.

Statistical Parameters	Carbon Fiber Composite		Glass Fiber Composite	
	*µ_m_* (cm^2^/g)	*µ* (cm^−1^)	*µ_m_* (cm^2^/g)	*µ* (cm^−1^)
Sum of squares for error (SSE)	0.001288	0.002421	0.001428	0.003477
R-square	0.96	0.9841	0.9527	0.9848
Adj R-square	0.9552	0.9821	0.947	0.9829
RMSE	0.007179	0.009841	0.007559	0.01179
